# Comprehensive Bee Pathogen Screening in Belgium Reveals *Crithidia mellificae* as a New Contributory Factor to Winter Mortality

**DOI:** 10.1371/journal.pone.0072443

**Published:** 2013-08-26

**Authors:** Jorgen Ravoet, Jafar Maharramov, Ivan Meeus, Lina De Smet, Tom Wenseleers, Guy Smagghe, Dirk C. de Graaf

**Affiliations:** 1 Laboratory of Zoophysiology, Faculty of Sciences, Ghent University, Ghent, Belgium; 2 Department of Crop Protection, Faculty of Bioscience Engineering, Ghent University, Ghent, Belgium; 3 Laboratory of Socioecology and Social Evolution, K.U.Leuven, Leuven, Belgium; Wuhan Bioengineering Institute, China

## Abstract

Since the last decade, unusually high honey bee colony losses have been reported mainly in North-America and Europe. Here, we report on a comprehensive bee pathogen screening in Belgium covering 363 bee colonies that were screened for 18 known disease-causing pathogens and correlate their incidence in summer with subsequent winter mortality. Our analyses demonstrate that, in addition to *Varroa destructor*, the presence of the trypanosomatid parasite *Crithidia mellificae* and the microsporidian parasite *Nosema ceranae* in summer are also predictive markers of winter mortality, with a negative synergy being observed between the two in terms of their effects on colony mortality. Furthermore, we document the first occurrence of a parasitizing phorid fly in Europe, identify a new fourth strain of Lake Sinai Virus (LSV), and confirm the presence of other little reported pathogens such as *Apicystis bombi,* Aphid Lethal Paralysis Virus (ALPV), *Spiroplasma apis*, *Spiroplasma melliferum* and *Varroa destructor* Macula-like Virus (VdMLV). Finally, we provide evidence that ALPV and VdMLV replicate in honey bees and show that viruses of the LSV complex and Black Queen Cell Virus tend to non-randomly co-occur together. We also noticed a significant correlation between the number of pathogen species and colony losses. Overall, our results contribute significantly to our understanding of honey bee diseases and the likely causes of their current decline in Europe.

## Introduction

Pollination is vital to the functioning of natural ecosystems, boosting the reproduction of wild plants, on which many other organisms depend. Likewise, many fruit, nut, vegetable and seed crops cultivated in an agricultural context depend on pollination. Honey bees (*Apis mellifera*) are considered the most economically valuable pollinators for crop monocultures worldwide [1].

However, over the last decade unusually high honey bee colony losses have been reported, mainly in North-America [2] and Europe [3]. There is a consensus nowadays that no single explanation can be given for these losses, and that there are several contributory factors to their decline, including pathogens, pesticides, nutrition and limited genetic diversity [2], [4], [5].

The ectoparasitic mite *Varroa destructor* is almost certainly a key player in causing the observed elevated colony losses [6]–[11]. This mite jumped from the Asian honey bee (*Apis cerana*) to the European honey bee (*Apis mellifera*) more than fifty years ago and has since become an almost cosmopolitan pest [12]. The mite weakens the bees by sucking hemolymph from both adult bees and pupae [13]. In addition, they can transmit many of the known honey bee viruses [14]–[18] and cause a reactivation of covert virus infections due to host immune suppression [19]. The mite destabilizes the within-host dynamics of viruses due to this immune suppression, which can then reach lethal levels [20]. Further, *V. destructor* and Deformed Wing Virus (DWV) will reduce the life span of winter bees, which can cause a colony collapse [21].

So far, only three viruses have been correlated with colony losses: DWV, Acute Bee Paralysis Virus (ABPV) and Israeli Acute Bee Paralysis Virus (IAPV). ABPV and IAPV are members of a complex of closely related Dicistroviridae [22]. IAPV was initially identified as a predictive marker for colony losses in the USA [23]. An expanded study could not confirm this result [5]. Moreover, a retrospective study revealed that this virus was already present before the first colony collapse disorders were ever reported [24]. In Europe, ABPV was linked with colony losses in Belgium [25], Germany [10] and Switzerland [26]. Furthermore, DWV has been linked to winter mortality in both Switzerland [26] and Germany [10].

The role of the Microsporidian fungus *Nosema ceranae*, another parasite that originates from the Asian honey bee [27], in causing colony collapse is still controversial. Sudden colony collapses in Spain were attributed to *N. ceranae* infection [28], [29], but these observations could not be confirmed by later independent studies in and outside Spain [2], [23], [30]–[32].

Recently, a prospective study revealed the presence of the little reported pathogens *Crithidia mellificae, Spiroplasma apis* and *Spiroplasma melliferum* in large-scale migratory beekeeping operations in the USA. Furthermore, the novel viruses (Aphid Lethal Paralysis Virus (ALPV) strain Brookings, Big Sioux River Virus (BSRV), Lake Sinai Virus (LSV) 1 and 2) and the phorid fly *Apocephalus borealis* were discovered as honey bee pathogens [33]. Earlier descriptions of spiroplasmas in honey bees go back to the early eighties [34], [35]. Also *C. mellificae* has been little studied since its first description in 1967 [36], even though the related *Crithidia bombi* is known to have serious effects on bumble bees, particularly under starvation conditions [37], [38]. The prevalence of these and other new pathogens and their potential correlation with winter losses in Europe, where no large-scale migratory commercial beekeeping occurs, is at present unknown.

In 2011, we performed an epidemiological study of the most common honey bee viruses in Belgium [39]. As shortly afterwards several neglected and new honey bee pathogens were described in the USA [33], we decided to re-examine these samples in order to type them for several of these other known honeybee pathogens. Based on this, we here provide the first molecular evidence for the presence of parasitic phorid flies in honey bee samples in the Palaearctic region, and demonstrate the presence of two *Spiroplasma* spp., *Apicystis bombi*, ALPV strain Brookings, *C. mellificae*, different LSV strains and *Varroa destructor* Macula-like Virus (VdMLV) in Belgian honey bees. In addition, we examine whether the presence of these pathogens in the summer can be used as a predictor of later winter mortality, and study possible associations in the prevalence of these pathogens.

## Materials and Methods

### Honey Bee Sampling and Preparation

For detailed description of the worker bee sampling procedure we refer to our previous paper [39]. In brief, in July 2011 around 30 bees were randomly sampled at the hive entrance of 363 colonies. RNA was extracted from 10 bees per colony for the molecular detection of pathogens. In addition, the natural *Varroa* drop was monitored by placing a sheet of paper under the open mesh floor during one week, and counting the mites in the laboratory. Optimization of the PCR for VdMLV was done on mites collected at the apiary of Ghent University, campus Sterre. This is a newly discovered virus in both mites and honey bees [40].

### MLPA Analysis

BeeDoctor [39], a ‘multiplex-ligation probe dependent amplification’ (MLPA) based method capable of detecting CBPV, DWV complex, ABPV complex, BQCV, SBPV and SBV, was expanded with probes to detect the positive and the negative strand each of BSRV, ALPV strain Brookings and viruses of the LSV complex (Table S1). Because of the high similarities of the two described LSV strains [33], we were unable to differentiate between them. This new prototype of BeeDoctor was used for screening purposes in the present study. All probes were synthesized by Integrated DNA Technologies (Leuven, Belgium). BeeDoctor analysis was performed as described before [39], starting from 1 µl RNA. All the MLPA reagents were obtained from MRC-Holland. The amplified MLPA products were analysed using 4% high resolution agarose gel electrophoresis.

### PCR Analysis

Five µl RNA (variable concentration) was retro-transcribed using random hexamer primers with the RevertAid™ First Strand cDNA Synthesis Kit (Thermo Scientific), according to the manufacturer’s instructions. All PCR reaction mixtures contained 2 µM of each primer; 1.5 mM MgCl_2_; 0.2 mM dNTP; 1.25 U Hotstar Taq DNA polymerase (Qiagen) and 1 µl cDNA product.

The primers used are shown in Table S1. Temperature cycles for slowly-evolving trypanosomatids and neogregarines were as described [39], but PCRs were performed in their uniplex mode. Samples that were positive for trypanosomatids were sequenced to confirm the presence of *C. mellificae*. Positive samples of neogregarines were subsequently re-analyzed with *A. bombi*-specific primers. Fifteen amplicons were sequenced for verification. Spiroplasmas were detected as described [41], based on the 16S ribosomal RNA sequence. Due to unspecific bands from *N. ceranae* rRNA around 700 bp, only universal *Spiroplasma* primers were used. Amplicons around 1 kb were extracted using the GeneJET Gel Extraction Kit (Thermo Scientific) and sequenced for *S. apis* and *S. melliferum* differentiation. For the differentiation of *Nosema apis* and *N. ceranae*, PCR conditions described by [42], [43] were used. Samples negative with these primers but positive for *Nosema* spore counting, were re-analyzed with primers specific for Microsporidia. A subset of the amplicons was sequenced for verification.

In order to detect the LSV strain 1 and 2, we followed the procedure described by [33]. However, when other strains appeared to be present we developed a PCR to detect a partial sequence of the Orf1 and RNA-dependent RNA polymerase genes of any known member of the LSV complex (strain 1, 2 and 3 at that time) using a degenerated primer set and the following cycling conditions: 94°C for 15 min; 94°C for 30 sec, 60°C for 30 sec, 72°C for 1 min; 35 cycles; final elongation 72°C for 10 min; hold at 4°C. Amplicons around 600 bp were extracted and sequenced. Temperature cycles for Microsporidia, Phoridae, ALPV and VdMLV were as described above, but with the annealing temperature set at respectively 60°C, 59°C, 60°C and 51°C. All PCR products were electrophoresed in 1.4% agarose gels, stained with ethidium bromide and visualised under UV light.

We developed a positive control for the Phoridae PCR and the MLPA-based detection of ALPV and BSRV by synthesizing 486-mer, 160-mer and 190-mer oligonucleotides respectively in a pIDTSmart vector (done by Integrated DNA Technologies). In other cases, the first positive sample detected in preliminary screenings served as a positive control.

### Cloning and Sequencing

Amplicons of ALPV strain Brookings, LSV complex, VdMLV and Phoridae were cloned into the pCR4 TOPO vector from TOPO TA Cloning Kit for sequencing (Invitrogen, USA) according to manufacturer’s instructions. The cloned inserts were sequenced on an ABI 3130XL platform using M13 primers after isolation of the plasmids with the GeneJet™ Plasmid Miniprep kit (Thermo Scientific).

DNA sequences obtained by direct sequencing of amplicons or by sequencing cloned PCR products were BLAST-searched at http://blast.ncbi.nlm.nih.gov/. Alignments of the LSV amplicons and strict consensus sequences (100% threshold) from LSV 1 (Genbank: HQ871931), LSV 2 (Genbank: HQ888865) and LSV 3 (Genbank: JQ480620) RNA polymerases and Orf1 genes were generated with Geneious 5.6.4.

### Nosema Spore Counting

We determined the *Nosema* spore levels in the extracts from 10 bees using light microscopy and a haemocytometer according to Cantwell [44]. This extract was diluted when necessary.

### Statistics

The multiple-kind lottery model of [45] was used to infer the theoretical distribution of pathogens in surviving and collapsed colonies. By use of the individual infection percentage of each pathogen (n = 16) the model calculates the expected pathogen distribution or the number of colonies infected with 0 to 16 pathogens. As described earlier [46], significant deviations between the observed and theoretically predicted pathogen distributions imply an interaction between different pathogens in this multi-pathogen host system. By means of a Pearson Chi-square test (*P*<0.05) with SPSS 21.0 we compared if the observed pathogen distribution differed from the theoretical distribution. The same approach was followed to infer which interaction between pathogen pairs occurred within this multi-pathogen host system.

Pathogen prevalence was correlated with winter mortality using a binomial generalized linear model with probit link function using function *glm* in package *stats* in *R* 2.16. This analysis was performed with a subset of the samples (229), since we excluded colonies for which the beekeepers did not provide any data on winter mortality, as well as colonies that had undergone queen supersedure. To select the most parsimonious model we used an exhaustive search based on the Akaike Information Criterion (AIC). This was done using R package *glmulti*, based on a set of predictor variables which either included all main effects (but excluding pathogens *S. apis*, CBPV and ABPV, since they occurred in fewer than 10 out of 229 colonies), or one which also considered possible first order interaction effects, and which included the pathogens which in a full main effects model had probit coefficients >0.2 (*N. ceranae, C. mellificae, V. destructor, S. melliferum* and BQCV) as well as DWV, which had been linked to winter mortality before [10], [26]. In addition, we also ran a model in which all main effects were included as well as a first and third order polynomial model in which the total number of detected pathogens was used as a predictor of winter mortality. Significance was assessed using Type III likelihood ratio tests using function Anova in R package *car*. In all cases, one-sided *p*-levels were used, since pathogens a priori are expected to increase colony mortality. The predictive power of our resulting models was assessed using function CVbinary in R package DAAG.

## Results

### Survey of Pathogens

An overview of the prevalences of the investigated pathogens is given in Table 1. The natural *Varroa destructor* drop ranged from 0 to more than 500 mites per week. A value equal to 0 does not necessarily imply that the colony is uninfected, only that the *Varroa* drop is below the detection limit. Within the boundaries, a prevalence of 93.7% (313/334) was found. *Nosema* spores were found in 75.2% (273/363) of the samples, and ranged from 10^5^ till 10^9^ per bee. PCR-based detection reveals 10.2% *N. apis* infection (37/363) and 92.6% *N. ceranae* infection (336/363), accounting for a total *Nosema* prevalence of 93.9% (341/363). Mixed infections, single *N. apis* and *N. ceranae* infection occurred in respectively 8.8% (32/363), 1.4% (5/363) and 83.7% (304/363) of the samples.

**Table 1:** Honey bee pathogen incidences.

Pathogen	Type	Prevalences			Associations	
		Overall	Surviving colonies*	Collapsed colonies*	Between pathogens	With winter losses*
ABPV	Dicistroviridae	3.3% (12/363)	3.3% (4/122)	3.7% (4/107)		No
ALPV	Dicistroviridae	56.2% (204/363)	59.0% (72/122)	54.2% (58/107)	*Nosema* spores (p = 0.011)	No
*Apicystis bombi*	Ophryocystidae	40.8% (148/363)	41.8% (51/122)	41.1% (44/107)		No
*Apocephalus borealis*	Phoridae	31.1% (118/363)	32.8% (40/122)	33.6% (36/107)		No
BQCV	Dicistroviridae	13.5% (49/363)	10.7% (13/122)	14.0% (15/107)	LSV complex (p = 0.009)	No
CBPV	Unclassified RNA virus	1.7% (6/363)	0.0% (0/122)	1.9% (2/107)		No
*Crithidia mellificae*	Trypanosomatidae	70.5% (256/363)	71.3% (87/122)	81.3% (87/107)		Yes (p = 0.03)
DWV	Iflaviridae	69.4% (252/363)	61.5% (75/122)	67.3% (72/107)		No
LSV complex	Unclassified RNA virus	14.6% (43/363)	17.2% (21/122)	15.0% (16/107)	BQCV (p = 0.009)	No
*Nosema apis*	Nosematidae	10.2% (37/363)	13.1% (16/122)	10.3% (11/107)		No
*Nosema ceranae*	Nosematidae	92.6% (336/363)	89.3% (109/122)	94.4% (101/107)	VdMLV (p<0.001)	No
*Nosema* spores	Nosematidae	75.2% (273/363)	71.3% (87/122)	72.9% (78/107)	ALPV (p = 0.011)	No
SBV	Iflaviridae	19.0% (69/363)	17.2% (21/122)	21.5% (23/107)		No
*Spiroplasma apis*	Spiroplasmataceae	0.3% (1/363)	0.0% (0/122)	0.0% (0/107)		No
*Spiroplasma melliferum*	Spiroplasmataceae	4.4% (16/363)	3.3% (4/122)	6.5% (7/107)		No
*Varroa destructor*	Varroidae	93.7% (313/334)	91.0% (111/122)	95.3% (102/107)		Yes (p = 0.07)
VdMLV	Tymoviridae	84.3% (306/363)	79.5% (97/122)	84.1% (90/107)	*N. ceranae*(p<0.001)	No

Prevalences of honey bee pathogens found in Belgian honey bee colonies, the relationships between these pathogens and the effect of the occurrence of each pathogen on colony winter losses.

* These data includes a subset of the samples (229), since 25% of the beekeepers did not provide data about winter losses of the monitored colonies.

Amplicons had an almost complete nucleotide similarity with sequences of *N. apis* (Genbank: U97150) or *N. ceranae* (Genbank: DQ486027).

While an ALPV strain and different LSV strains were fairly abundant, with prevalences of 56.2% (204/363) and 14.6% (43/363) in our studied colonies, BSRV could not be detected. ALPV amplicons shared 97% nucleotide identity with two strains isolated from honey bees (Genbank: HQ871932; JX045858) and 89% with the canonical ALPV sequence (Genbank: AF536531). At the amino acid level, our isolates (Genbank: KC880119) appeared to be identical to ALPV strain Brooking and 99% identical to a Spanish strain (Genbank: JX045858), caused by one substitution of valine to isoleucine. Moreover, we could show that ALPV and VdMLV are replicating in honey bees by demonstrating the presence of their negative strand intermediate, a marker for replication of positive sense single stranded RNA viruses, by a strand specific MLPA reaction. Surprisingly, VdMLV was detected in the majority of our bee samples (84.3%; 306/363). The Belgian strain (Genbank: KC880120) showed high sequence homology (97% on nucleotide level, 99% on amino acid level) with a French strain (Genbank: HQ916350).

The spiroplasmas *S. apis* and *S. melliferum* were found only in respectively 0.3% (1/363) and 4.4% (16/363) of the tested samples. One sequence appeared 100% identical to the *S. apis* strain ATCC 33834 (Genbank: GU993267); all others matched to *S. melliferum* IPMB4A (Genbank: JQ347516) (4 sequences with 100% identity and 6 sequences with only a single nucleotide substitution).

The little reported trypanosomatid *C. mellificae* was found in 70.5% (256/363) of the samples. The amplicons showed 100% sequence identity with a partial sequence of the small subunit ribosomal RNA of *C. mellificae* (Genbank: AB745488). We also found molecular evidence that the neogregarine *A. bombi*, primarily known as a bumble bee parasite [47], was present in 40.8% (148/363) of our samples. The 15 sequenced amplicons showed 100% identity with a partial small subunit ribosomal RNA sequence of *A. bombi* (Genbank: FN546182).

Unexpectedly, we were also able to demonstrate the molecular presence of phorid flies in 31.1% (118/363) of the samples. These amplicons fully matched a partial *A. borealis* 18S ribosomal RNA sequence (Genbank: JF808447).

### Identification of the LSV Strains

In order to determine which LSV strains we had found by MLPA, we re-investigated the positive samples by PCR using the primers specific for LSV 1 and LSV 2 [33]. These specific primers did not work on our samples and therefore a degenerated primer set was developed. The sequence of the amplicons generated with the degenerated primer set revealed one sample (Genbank: KC880123) with high resemblance to a known strain (Genbank: JQ480620) (96% nucleotide and 94% amino acid identity with LSV 3, a third LSV type that was described in the meanwhile [48]), while others gave only moderate similarity to any of them (Genbank: KC880121, KC880122, KC880124-KC880126). Amplicons from six apiaries had the same trimmed sequence, which aligned very well with the consensus sequence of the RNA-dependent RNA polymerases of the three different strains (Figure S1). We designated this sequence representative for a new fourth strain of LSV (Genbank: JX878492). The LSV Orf1 sequences showed a high degree of sequence divergence (data not shown) but the majority of the conserved Orf1 amino acids were also retrieved in LSV 4 and our other sequences.

### Effect of Pathogens on Colony Winter Losses

Overall, 46.5% of the sampled colonies were reported to be lost over the winter of 2011–2012. Combined with our data on the prevalence of 16 known honeybee pathogens in these colonies in summer (July 2011), including several little reported ones detected in the present paper, but also the more common viruses detected previously in these samples [39], we decided to test whether these winter losses could be predicted on the bases of the presence or absence of these pathogens (Table 1).

**Figure 1:**
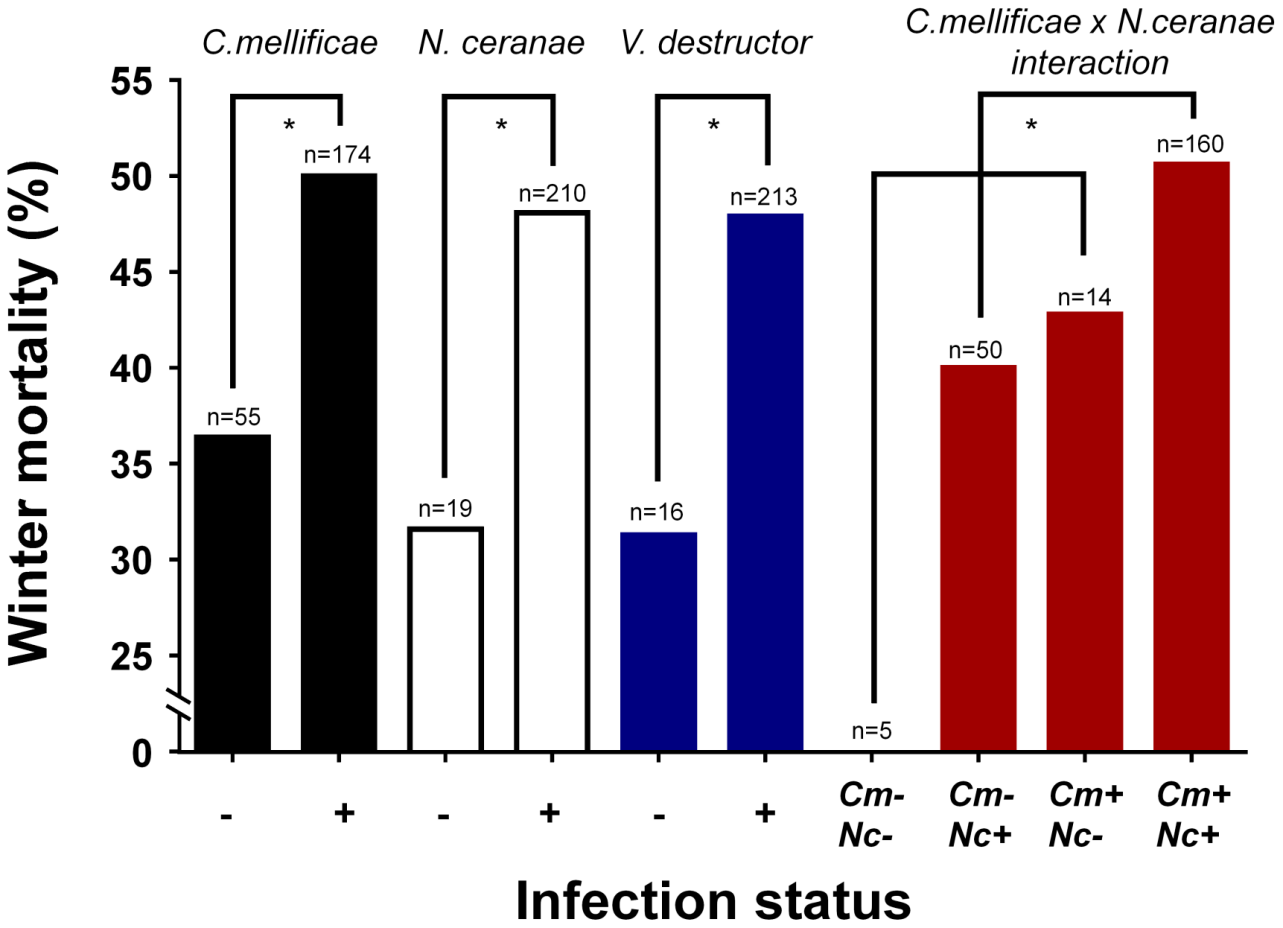
Effect of *Crithidia mellificae, Nosema ceranae and Varroa destructor* on honey bee colony winter losses. The presence of *C. mellificae*, *N. ceranae* and *V. destructor* in summer all increase later winter mortality (binomial probit model, Table 2b, p = 0.01, 0.03 and 0.07, respectively). In addition, there is a synergistic effect of *C. mellificae* and *N. ceranae* on winter mortality (Table 2b, p = 0.03). Cm = *C. mellificae*, Nc = *N. ceranae*.

**Figure 2:**
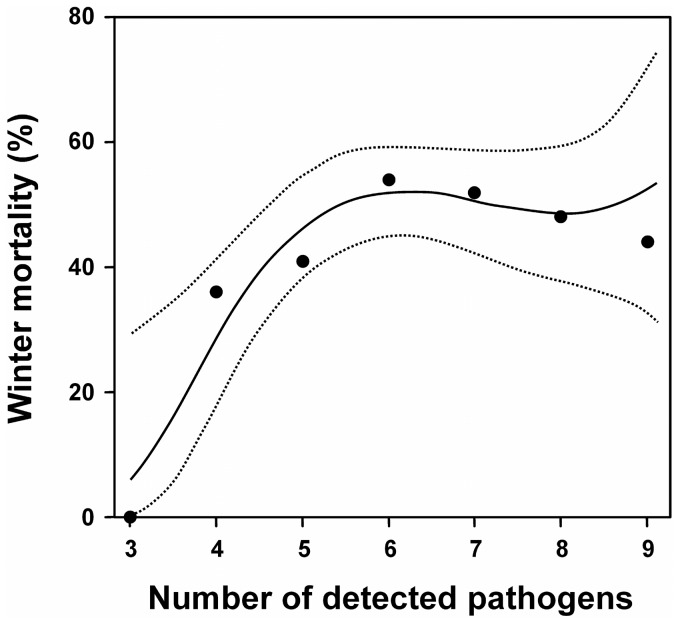
Effect of the number of detected pathogens on winter mortality, based on a third order binomial probit model. The predicted winter mortality goes up markedly when the number of detected pathogens increase from 3 to 6 (from 5.9% to 52%), but then stabilizes around 50% when colonies have higher total numbers of pathogens.

Based on a probit binomial model in which only main effects were considered, an exhaustive model search showed that *V. destructor* and *C. mellificae* contributed most to explaining winter mortality (AIC = 317.21) (*C. mellificae*: p = 0.03, marginal odds ratio = 1.3; *V. destructor*: p = 0.07, marginal odds ratio = 1.3, Table 2a). Nevertheless, if we also included first order interaction effects and carried out an exhaustive search we obtained a model with slightly better explanatory power (AIC = 316.11). *C. mellificae*, *N. ceranae*, *V. destru*ctor and as well as the interaction effect *C. mellificae* × *N. ceranae*, significantly contributed to explaining winter mortality in this model (p = 0.01, 0.02, 0.07 and 0.03, respectively; Table 2 and Figure 1). The significant interaction effect was due a negative synergy between *C. mellificae* and *N. ceranae* on winter mortality (Figure 1). It means that the combination of both pathogens has a lesser output than the sum of each pathogen. Nevertheless, a clear enhancing effect can still be observed. Based on this model, the accuracy of the prediction of whether a colony would die or not in the winter was 55% using internal estimates, or 52% using cross-validation. Overall, higher numbers of detected pathogens in summer also resulted in a significantly increased winter mortality, as shown by a first order probit model (AIC = 316.93, p = 0.03). In addition, the use of a third order probit model further increased the accuracy of the fit to the data (AIC = 316.12), and resulted in a significantly positive first order effect (p = 0.02) and a significantly negative second order effect (p = 0.03) of the number of detected pathogens on winter mortality (Figure 2). When the amount of detected pathogens increases from 3 to 6 (from 5.9% to 52%), the predicted winter mortality goes up markedly but stabilizes around 50% at higher numbers of pathogen species.

**Table 2:** Effects of the screened pathogens in summer on the observed honeybee winter mortality, based on probit models and exhaustive model searches in which the Akaike Information Criterion (AIC) was minimized.

Pathogen	Probit coefficients	Marginal odds ratios	LR *χ* ^2^	*p* value
**(a) Best model based on main effects only** a **(AIC 317.21)**				
Intercept	−0.82			
*Crithidia mellificae*	0.37	1.3	3.57	0.03
*Varroa destructor*	0.49	1.4	2.11	0.07
**(b) Best model based on most important main effects and their 1st order interactions** b **(AIC 316.11)**				
Intercept	−5.70			
*Crithidia mellificae*	5.09	1.3	6.16	0.01
*Nosema ceranae*	4.97	1.4	4.88	0.03
*Varroa destructor*	0.49	1.4	2.10	0.07
*Crithidia mellificae* x *Nosema ceranae*	−4.80	0.8	3.37	0.03

a Based on an exhaustive search with the following set of predictor variables: presence or absence of N. apis, N. ceranae, C. mellificae, A. bombi, S. melliferum, A. borealis, ALPV, DWV, BQCV, SBV, LSV, VDMLV, V. destructor as well as the natural V. destructor drop and Nosema spore load.

b Based on an exhaustive search, including all pathogens which occurred in more than 10 out of 229 colonies and which in a full main effects model had probit coefficients >0.2 (N. ceranae, C. mellificae, V. destructor, S. melliferum and BQCV) and DWV, which has been linked to winter mortality before [10], [26], as well as their first order interaction effects.

### Relationships between Pathogens

**Figure 3:**
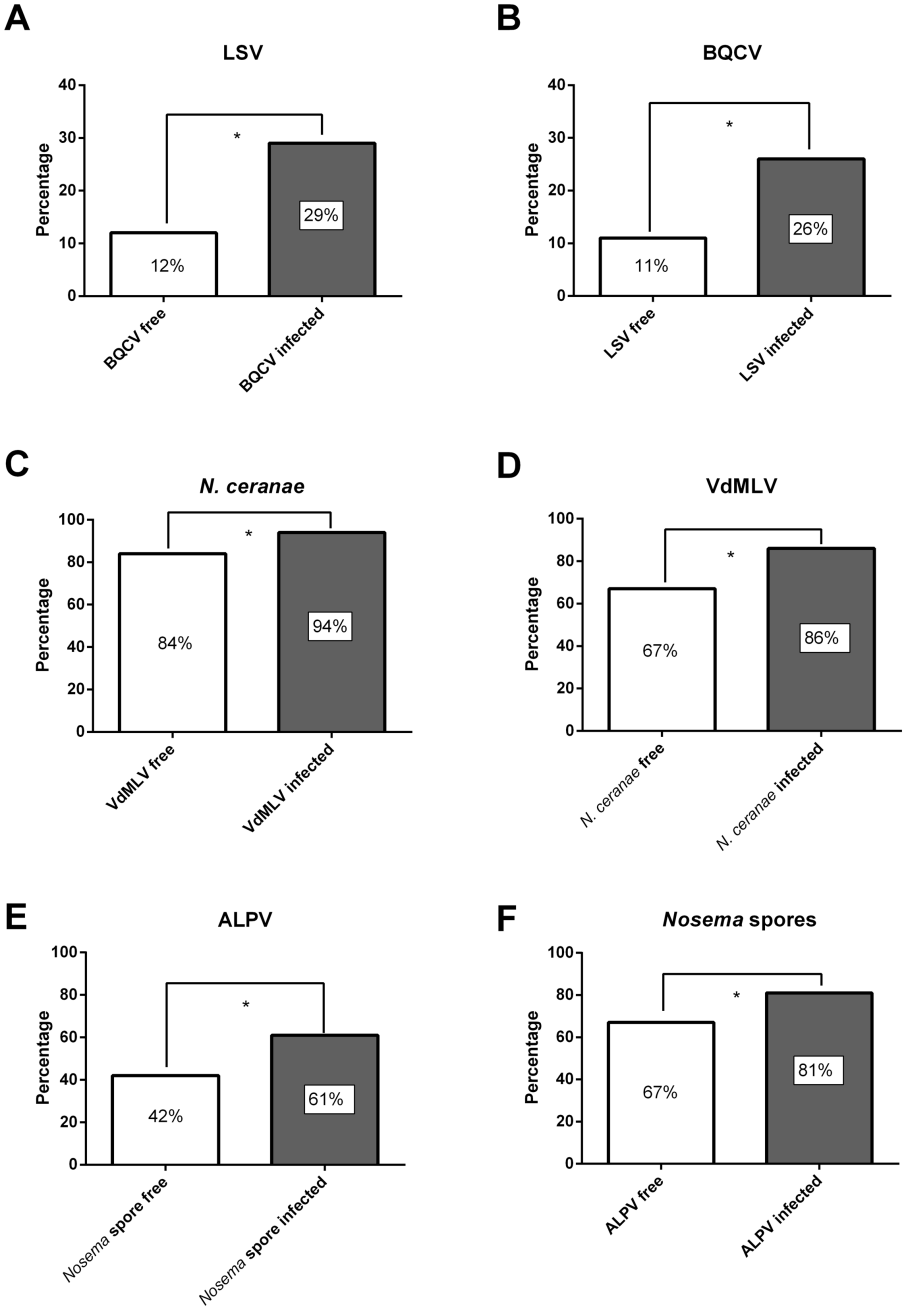
Graphical representation of significant pathogen correlations. LSV complex is significantly associated with BQCV (p = 0.009) (**A** and **B**), VdMLV with *N. ceranae* (p<0.001) (**C** and **D**) and ALPV strain Brookings with *Nosema* spores (p = 0.011) (**E** and **F**).

As determined by a Pearson Chi-square test, we found evidence for positive associations between different pathogens (p<0.05). LSV was significantly associated with BQCV (χ^2^ = 9.41, df = 2, p = 0.009), ALPV with *Nosema* spores (χ^2^ = 9,087, df = 2, p = 0.011) and VdMLV with *N. ceranae* (χ^2^ = 28.067, df = 2, p<0.001). These pathogen associations are presented graphically in Figure 3.

## Discussion

Overall, our data represent among the most comprehensive prevalence studies of honey bee pathogens carried out to date in Europe. The recent discovery of new bee viruses and neglected parasites in several countries highlighted the narrow window of pathogens that are the subject of many monitoring programs. As a result, we decided to re-investigate samples from July 2011 and statistically analyze whether the detected pathogens in summer had any effect on the winter mortality.

Our analysis confirmed the importance of *V. destructor* in summer as a marker for colony collapses [7] (Figure 1). Importantly, our analysis also demonstrated a large effect of the occurrence of *C. mellificae* in summer on later winter losses, even enhanced through *N. ceranae* co-infection (Figure 1). The protozoan *C. mellificae* has been ignored for a long time, but the current data highlight it as a new putative key player in honey bee colony declines. This trypanosome has probably a cosmopolitan distribution since it has been reported in Australia [36], China [49], France [50], Japan [51], Switzerland [52] and USA [33]. Besides, the related *C. bombi*, also reported from Asian honey bees [53], has serious effects on the survival of bumble bees under stress conditions [37]. Recently, complex dynamic immune responses to *C. mellificae* infection were reported, with a distinct response when individuals were infected with *C. mellificae* and *N. ceranae* simultaneously [54]. In addition, an association between both pathogens was reported in the USA [33]. Possibly, the controversial role of *N. ceranae* [23], [29], [55] might be explained by the synergistic effect of *N. ceranae* and *C. mellificae* on colony mortality. We also observed a significant correlation between the number of detected pathogens and colony losses, as was likewise reported in the USA [5]. Collapsing colonies, induced by e.g. *V. destructor* and *C. mellificae*, are probably more vulnerable to a diverse set of parasites [48], which elucidate this correlation. Moreover, it appeared that several pathogens can act synergistically and eventually cause a collapse of the honey bee colony [48]. The outcome of these pathogen interactions can vary between regions [48], probably because of the multifactorial origin of colony losses and the interplay between different stressors.

Additionally, our results confirm that Lake Sinai Viruses are a viral complex (Figure S1). Diverse viral sequences are reported in the USA [33], [48] and Spain [56]. We could also confirm the presence of one known American LSV strain in Belgium, namely LSV 3. Another strain, designated LSV 4 (Genbank: JX878492), was retrieved in several independent samples. An ALPV strain was detected for the first time in Belgium. This virus was also detected in American [33] and Spanish honey bees [56]. Remarkable was its rather high incidence in the present study (56.2%; 204/363), akin to similar observations in different regions in the USA [33]. We could detect the presence of the ALPV negative strand intermediate, demonstrating that it is a replicating virus in honey bees. It is associated with the presence of *Nosema* spores, being indicative for a common oral transmission route. Another less known virus, VdMLV, was suggested to be a virus of *V. destructor*, which can be transmitted to honey bees [57]. Surprisingly, our study indicates a high prevalence in honey bees and a correlation with *N. ceranae*. The impact of this virus on honey bees remains unclear, but might well be significant since it replicates in honey bees.

The bacteria, *S. apis* and *S. melliferum*, are known as honey bee pathogens for a long time [34], [35], including Asian honey bees [58]. They seem to be uncommon in honey bees, with a sudden incidence in the summer [33] which may be related to transmission via flowers.

Another unexpected discovery was the detection of phorid flies. Since the found amplicons had a 100% nucleotide similarity, we have strong molecular evidence that *A. borealis* or a similar phorid fly also infects honey bees outside the USA. To our knowledge this is the first description of a parasitizing phorid fly in honey bee samples in a Palaearctic region. This phorid fly was recently described as a new honey bee pathogen which alters the host behaviour by hive abandonment, eventually causing death [59].

Besides viruses, bacteria and fungi, honey bees can also be parasitized by neogregarines. Our study revealed a high prevalence of *Apicystis bombi* in honey bees. This parasite is believed to be highly virulent in bumble bee spring queens, but re-emerges later on in worker bumble bees [46]. However, real empirical data is missing to describe the pathology of *A. bombi*. After its detection in honey bees in Finland [47], *A. bombi* was also reported in honey bees in Japan [51] and Argentina [60].

The presence in Argentina is probably induced by spillover from invasive *Bombus terrestris* [61], an introduced pollinator outside the West Palaearctic area [62]. The high prevalence (40.8%; 148/363) of *A. bombi*, without correlation with winter losses, indicates that it is not highly virulent in honey bees. Surprisingly, unbiased molecular studies in the USA did not report the occurrence of this pathogen [23], [48].

## Conclusions

Colony winter losses in Belgium seem to be associated with (1) *V. destructor* and (2) the detection of *C. mellificae* and *N. ceranae* in summer, with an enhancing effect on colony mortality being observed between the latter two. Thus, the present study not only extends the number of pathogens bees are exposed to in Europe, but also assigned the trypanosomid parasite *C. mellificae* as a new contributory factor to explain winter losses, in addition to the parasitic mite *V. destructor* and the microsporidian parasite *N. ceranae*. Moreover, the present study describes the occurrence of 6 new pathogens in Belgian honey bee: ALPV strain Brookings, VdMLV, viruses of the LSV complex, *S. melliferum*, *A. bombi* and *A. borealis*. This phorid fly and *S. melliferum* were hitherto not reported as honey bee pathogens in Europe before. From LSV a new fourth strain was discovered. Screening for negative strand intermediate of these viruses demonstrated replication of ALPV and VdMLV in honey bees, which had never been demonstrated before. Furthermore, we found associations between viruses of the LSV complex and BQCV, between VdMLV and *N. ceranae*, and between an ALPV strain and *Nosema* spores. The latter might indicate a common oral route of transmission. From our data it seems advisory to look at a broader range of pathogens in nationwide monitoring programs.

## Supporting Information

Figure S1 **Sequence variability of Lake Sinai Virus RNA-dependent RNA polymerase.** Amino acid alignment of a consensus sequence (generated from LSV 1, 2 and 3) with known and new Lake Sinai Virus RNA-dependent RNA polymerase sequences.
(TIF)

Table S1 Primers and MLPA probes used in this study. Half-probes used for detecting different honey bee viruses or virus species complexes through RT-MLPA and primers used for detecting honey bee viruses or other pathogens. Each RPO probe is 5′- phosphorylated (indicated by ^P−^) to permit ligation of the 5′ end of the RPO to the 3′ end of the LPO. The PCR sequence tags on each halfprobe are in lower-case letters, the non-specific stuffer sequences (for generating PCR products with pre-determined sizes) are shown in upper-case letters and the target-specific sequences are shown in underlined upper-case letters.
(DOCX)
