# Supplementary figures and images for: Comprehensive Bee Pathogen Screening in Belgium Reveals Crithidia mellificae as a New Contributory Factor to Winter Mortality
Source: PLoS One. 2013 Aug 26;8(8):e72443. doi: 10.1371/journal.pone.0072443 (PMC3753275; doi:10.1371/journal.pone.0072443)

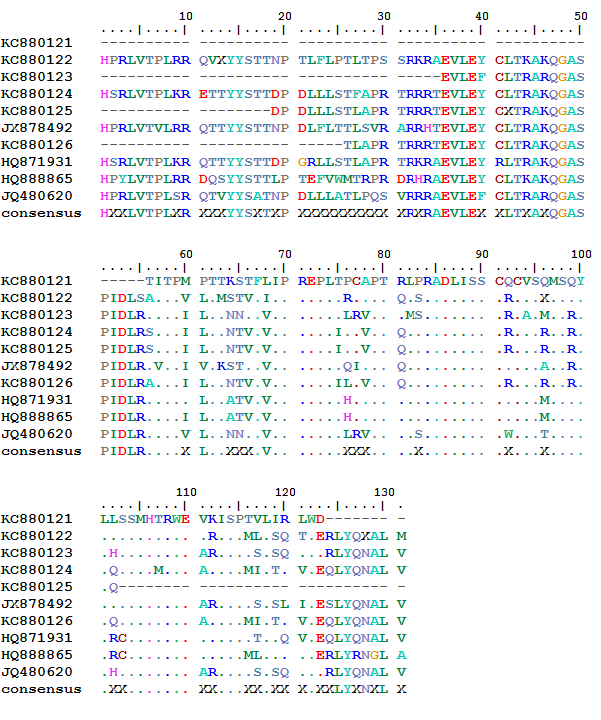

Supplement: Figure S1 — Sequence variability of Lake Sinai Virus RNA-dependent RNA polymerase. Amino acid alignment of a consensus sequence (generated from LSV 1, 2 and 3) with known and new Lake Sinai Virus RNA-dependent RNA polymerase sequences. (TIF) [file pone.0072443.s001.tif]
