# Supplementary material for: Comprehensive Bee Pathogen Screening in Belgium Reveals Crithidia mellificae as a New Contributory Factor to Winter Mortality
Source: PLoS One. 2013 Aug 26;8(8):e72443. doi: 10.1371/journal.pone.0072443 (PMC3753275; doi:10.1371/journal.pone.0072443)
Supplement: Table S1 — Primers and MLPA probes used in this study. Half-probes used for detecting different honey bee viruses or virus species complexes through RT-MLPA and primers used for detecting honey bee viruses or other pathogens. Each RPO probe is 5′- phosphorylated (indicated by P−) to permit ligation of the 5′ end of the RPO to the 3′ end of the LPO. The PCR sequence tags on each halfprobe are in lower-case letters, the non-specific stuffer sequences (for generating PCR products with pre-determined sizes) are shown in upper-case letters and the target-specific sequences are shown in underlined upper-case letters. (DOCX) [file pone.0072443.s002.docx]

**Table S1.** Primers and MLPA probes used in this study.

| **Target** | **Primers** | **Sequence (5' - 3')** | **Size (bp)** | **Reference** |
| --- | --- | --- | --- | --- |
| **MLPA detection** |  |  |  |  |
| ALPV-Br  (- strand) | MLPA_ALPV_rep_LPO | gggttccctaagggttggaTCCGTGGATTTATCATGCATAGCCAGTTCGGTTAATCC | 119 | This work |
|  | ALPV_rep_RPO | ^P­^GCCGCTGATTGTGTCAACACAGATACGTAGAGGTAGTTGtctagattggatcttgctggcac |  |  |
|  | RT_ALPV_rep | CCTAACTGGGTACGTGTTGG |  |  |
| ALPV-Br  (+ strand) | MLPA_ALPV_LPO | gggttccctaagggttggaTCTGACCTTTCACATCTGGACAGCCAACTACCTCTACGTATCTGTGTTGACACAATCAGC | 160 | This work |
|  | MLPA_ALPV_RPO | ^P­^GGCGGATTAACCGAACTGGCTATGCATGATAAAGTACAAGCCCGTTCAGCACCTGGGTtctagattggatcttgctggcac |  |  |
|  | RT_ALPV_new | TCATCTTAGACCTCCATTTAGAATCC |  |  |
| BSRV | BSRV-LPO | gggttccctaagggttggaGTGAGCAGTCAGGTGGCGTGATACGTGGTGTTTTTGATGACCTTGATCGAGTTCCAAAAGCACTGAGTGGCATG | 190 | This work |
|  | BSRV-RPO | ^P­^GAGAGTATGTGGAAACGCATAGACTCCGTGTCACTGAAAATATCAGCTATGCCGGACAGGGCGTGCGCGTTGAAtctagattggatcttgctggcac |  |  |
|  | RT_BSRV | CCATCCAATTCCATAGTACAGTTG |  |  |
| LSV complex | LSV-LPO | gggttccctaagggttggaGACTCCCAGCTGGACCGCTACGAAATGCGCGTATCCTCGTGCGGACCTCATTTCTTCATGTCAGTGT | 175 | This work |
|  | LSV-RPO | ^P­^GTGAGCATGATGAGTCAATACCTACGGTGTTCCGGGGATGGGGGCCGGGTGAGGAAAGCTGGCTGATCTAGATTGGATCTTGCTGGCACtctagattggatcttgctggcac |  |  |
|  | RT_LSV | CCACCGAGTGTGCATGG |  |  |
| **PCR detection** |  |  |  |  |
| ALPV-Br | ALP-Br-F-2936 | AACGTCGTATGCTACGATGAACTCG | 464 | [1] |
|  | ALP-Br-R-3400 | GGGTTAAATTCAATTCCAGTACCACGG |  |  |
| *A. bombi* | ApBF1 | CGTACTGCCCTGAATACTCCAG | ~ 511 | [2] |
|  | ApUR2 | TTTCTCATTCTTCAGATGATTTGG |  |  |
| *A. borealis* | Phorid_rRNA 1F | GTACACCTATACATTGGGTTCGTACATTAC | 486 | [1] |
|  | Phorid_rRNA 1R | GAGRGCCATAAAAGTAGCTACACC |  |  |
| LSV complex | LSVdeg-F | GCCWCGRYTGTTGGTYCCCCC | 578 | This work |
|  | LSVdeg-R | GAGGTGGCGGCGCSAGATAAAGT |  |  |
| LSV 1 | LSV1-F-2294 | TTATCTCGCGCCGCCACCTC | 672 | [1] |
|  | LSV1-R-2966 | ATCGCCGCTGCAACGTGACC |  |  |
| LSV 2 | LSV2-F-3954 | CGGCCGGTCTAGCGTGGTTG | 558 | [1] |
|  | LSV2-R-4512 | TGGCAAGCTGTGACGAATCCCT |  |  |
| Neogregarines | NeoF: | CCAGCATGGAATAACATGTAAGG | 258 | [2] |
|  | NeoR: | GACAGCTTCCAATCTCTAGTCG |  |  |
| Microsporidia | V1F | CACCAGGTTGATTCTGCCTGAC | ~ 406 | [3] |
|  | 530R | CCGCGGCTGCTGGCAC |  | [4] |
| *Nosema apis* | Napis-sense | CCATTGCCGGATAAGAGAGT | 269 | [5] |
|  | Napis-antisense | CCACCAAAAACTCCCAAGAG |  |  |
| *Nosema ceranae* | NceranaeF | CGGATAAAAGAGTCCGTTACC | 250 | [6] |
|  | NceranaeR | TGAGCAGGGTTCTAGGGAT |  |  |
| *Spiroplasma* spp. | BS1-F | AAGTCGAACGGGGTGCTT | 976 | [7] |
|  | BS1-R | TGCACCACCTGTCTCAATGT |  |  |
| Trypanosomatids | SEF | CTTTTGGTCGGTGGAGTGAT | 406 | [2] |
|  | SER | GGACGTAATCGGCACAGTTT |  |  |
| VdMLV | VdMLV-F | ATCCCTTTTCAGTTCGCT | 438 | [8] |
|  | VdMLV-R | AGAAGAGACTTCAAGGAC |  |  |

**Reference List**

1. Runckel C, Flenniken ML, Engel JC, Ruby JG, Ganem D, et al. (2011) Temporal Analysis of the Honey Bee Microbiome Reveals Four Novel Viruses and Seasonal Prevalence of Known Viruses, Nosema, and Crithidia. Plos One 6: e20656.

2. Meeus I, de Graaf DC, Jans K, Smagghe G (2010) Multiplex PCR detection of slowly-evolving trypanosomatids and neogregarines in bumblebees using broad-range primers. Journal of applied microbiology 109: 107-115.

3. Vossbrinck CR, Woese CR (1986) Eukaryotic Ribosomes That Lack A 5.8S RNA. Nature 320: 287-288.

4. Baker MD, Vossbrinck CR, Didier ES, Maddox JV, Shadduck JA (1995) Small-Subunit Ribosomal DNA Phylogeny of Various Microsporidia with Emphasis on Aids-Related Forms. J Eukaryot Microbiol 42: 564-570.

5. Chen Y, Evans JD, Zhou L, Boncristiani H, Kimura K, et al. (2009) Asymmetrical coexistence of Nosema ceranae and Nosema apis in honey bees. J Invertebr Pathol 101: 204-209.

6. Chen Y, Evans JD, Smith IB, Pettis JS (2008) Nosema ceranae is a long-present and wide-spread microsporidian infection of the European honey bee (Apis mellifera) in the United States. J Invertebr Pathol 97: 186-188.

7. Meeus I, Vercruysse V, Smagghe G (2012) Molecular detection of Spiroplasma apis and Spiroplasma melliferum in bees. J Invertebr Pathol 109: 172-174.

8. Gauthier L, Ravallec M, Tournaire M, Cousserans F, Bergoin M, et al. (2011) Viruses Associated with Ovarian Degeneration in Apis mellifera L. Queens. Plos One 6: e16217.
